# Dystrophin Dp71 and the Neuropathophysiology of Duchenne Muscular Dystrophy

**DOI:** 10.1007/s12035-019-01845-w

**Published:** 2019-12-13

**Authors:** Michael Naidoo, Karen Anthony

**Affiliations:** Centre for Physical Activity and Life Sciences, Faculty of Arts, Science and Technology, University of Northampton, University Drive, Northampton, Northamptonshire NN1 5PH UK

**Keywords:** Duchenne muscular dystrophy, Dystrophin, Dp71, Apo-dystrophin-1, Neurodevelopment

## Abstract

Duchenne muscular dystrophy (DMD) is caused by frameshift mutations in the *DMD* gene that prevent the body-wide translation of its protein product, dystrophin. Besides a severe muscle phenotype, cognitive impairment and neuropsychiatric symptoms are prevalent. Dystrophin protein 71 (Dp71) is the major *DMD* gene product expressed in the brain and mutations affecting its expression are associated with the DMD neuropsychiatric syndrome. As with dystrophin in muscle, Dp71 localises to dystrophin-associated protein complexes in the brain. However, unlike in skeletal muscle; in the brain, Dp71 is alternatively spliced to produce many isoforms with differential subcellular localisations and diverse cellular functions. These include neuronal differentiation, adhesion, cell division and excitatory synapse organisation as well as nuclear functions such as nuclear scaffolding and DNA repair. In this review, we first describe brain involvement in DMD and the abnormalities observed in the DMD brain. We then review the gene expression, RNA processing and functions of Dp71. We review genotype-phenotype correlations and discuss emerging cellular/tissue evidence for the involvement of Dp71 in the neuropathophysiology of DMD. The literature suggests changes observed in the DMD brain are neurodevelopmental in origin and that their risk and severity is associated with a cumulative loss of distal *DMD* gene products such as Dp71. The high risk of neuropsychiatric syndromes in Duchenne patients warrants early intervention to achieve the best possible quality of life. Unravelling the function and pathophysiological significance of dystrophin in the brain has become a high research priority to inform the development of brain-targeting treatments for Duchenne.

## Introduction

The X-linked neuromuscular disorder, Duchenne muscular dystrophy (DMD), is one of the most common fatal genetic disorders diagnosed in childhood. It is caused by frameshift mutations in the *DMD* gene that prevent the body-wide translation of its protein product, dystrophin. Although characterised by the progressive loss of muscle strength and function [1], cognitive impairment and neuropsychiatric symptoms are also prevalent. Mounting evidence links these symptoms to the loss of dystrophin in the brain.

Unlike skeletal muscle, the central nervous system (CNS) sees the expression of a large variety of *DMD* transcripts implicated in diverse cellular processes [2]. Dystrophin protein variants (Dp) are named based on their length in kilodaltons and are produced through unique promoter usage, alternative splicing and/or alternative polyadenylation signals. The most predominant in the brain is the Dp71 variant expressed in neurones and glia, except during foetal development where Dp140, also expressed throughout the CNS, dominates [3, 4]. Distal *DMD* mutations affecting the expression of these shorter variants are linked to cognitive impairment [5–11]. Dp140 and Dp71 are both heavily implicated. The risk and severity of cognitive disability are associated with a cumulative loss of distal *DMD* gene products [5, 12]. Thus, it is not entirely clear which, if any, single *DMD* gene product is responsible for the CNS phenotype. There is limited information on Dp140, likely due to its restricted expression pattern. In contrast, our knowledge of Dp71 has expanded rapidly, there are mouse models devoid of Dp71 and the literature has become complex. In light of this and the fact that 100% of individuals with mutations affecting Dp71 have intellectual disability [5, 6, 8, 9, 11], we limit the focus of this review to Dp71.

**Fig. 1:**
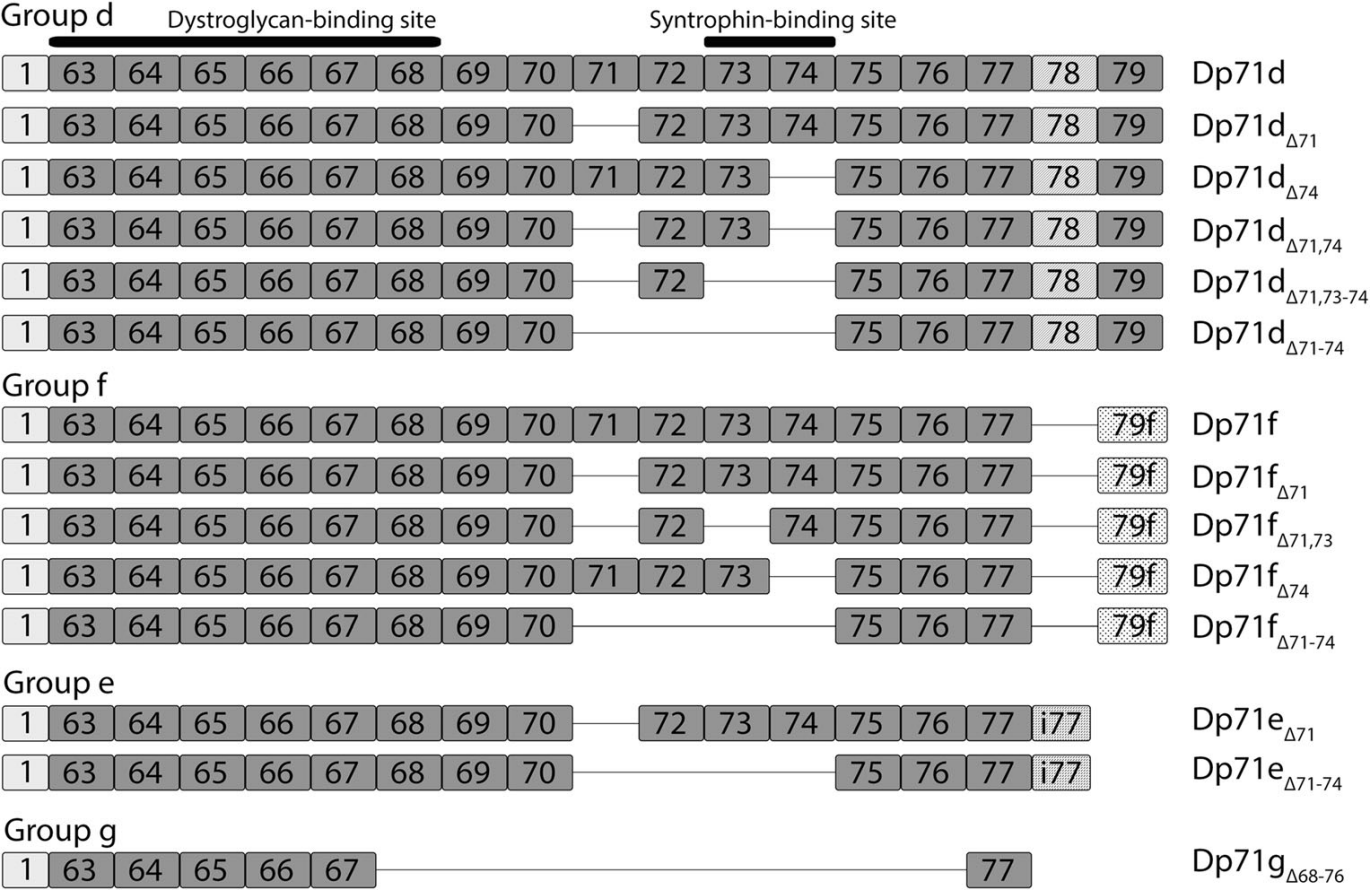
Dp71 isoforms and preferred nomenclature. Dp71 splice isoforms are grouped according to their C-terminus. The d group contain exons 78 and 79, the f group lacks exon 78 and has an alternative exon 79 (79f), the e group contains part of intron 77 (i77) and lacks exons 78 and 79 and the g group isoform has a stop codon in exon 77. Their differential C-termini are illustrated and the location of dystroglycan and syntrophin-binding sites are indicated

As many as 14 isoforms of Dp71 have now been described (Fig. 1) and our understanding of the function(s) of Dp71 has increased. Here, we provide a detailed update on Dp71 and review the accumulating evidence linking its loss to the neuropathophysiology of Duchenne. We first describe the ‘DMD neuropsychiatric syndrome’ coined by Ricotti [13] and the abnormalities observed in the DMD brain. We then review the gene expression and RNA processing of Dp71 and its function(s) and discuss how the absence and/or alteration of Dp71 likely contributes to the neuropathophysiology of DMD.

## The Duchenne Brain: Human and Animal Models

### Emotional, Behavioural and Neurodevelopmental Observations

Whilst most individuals with DMD are not intellectually disabled, the risk of cognitive impairment is higher than for the general population. Full-scale intelligent quotients (FSIQ) are consistently reported at one standard deviation below the normal population mean [13, 14] and levels of intellectual disability within Duchenne cohorts vary between approximately 19 and 35% (Table 1, [13–15]). Patients with distal *DMD* mutations affecting the expression of all *DMD* gene products have lower FSIQ scores than those with mutations that result in only the absence of full-length dystrophin [5]. Patients lacking Dp140 and/or Dp71 have a particularly high incidence of neurodevelopmental disorders [13]. There is a reported increase in epilepsy in patients with Duchenne compared with the general population (Table 1) and other brain-related comorbidities such as attention deficit hyperactivity disorder (ADHD) and obsessive compulsive disorder (OCD) are more prevalent in patients who have both DMD and epilepsy compared with non-epilepsy Duchenne patients [17]. Internalising disorders are reported in around a third of patients [13, 16] with depression, anxiety and OCD all having a higher prevalence than for the general paediatric population (Table 1). Externalising problems have been reported through parent reports in 15% of DMD patients [13], and other studies reveal a diagnosis of ADHD in up to 32% of DMD cohorts [10, 13, 28].

**Table 1:** Neurological comorbidities in DMD patients

Comorbidity	Approximate prevalence (%)	Reference(s)
Intellectual disability	19–35	[14–16]
Epilepsy	2–12	[17–20]
Autism	20	[13, 21–23]
Internalising problems	24–34	[13, 16]
Depression	17–27	[24–26]
Anxiety	24–29	[16, 25–27]
OCD	5–14	[16, 27, 28]
Externalising problems	15	[13]
ADHD	12–32	[10, 13, 28]
Reading disability	40–50	[29–32]

In addition to the comorbidities listed in Table 1, memory deficits are widely acknowledged with impairments in short-term working memory particularly apparent (e.g. story recall, digit span and auditory comprehension) [33–38]. Together with a high occurrence of reading disabilities (40–50%, Table 1) and speech delay [39], these deficits likely affect academic achievement. In the study by Ricotti [13], over a third of boys presented with at least two neurological comorbidities and Battini et al. [34] show that cognitive impairment is apparent even in patients without intellectual disability. Individuals with the similarly progressive disorder, spinal muscular atrophy, do not show the reading and learning disabilities described for DMD suggesting that cognitive impairment in DMD does not depend on motor disability [40]. The breadth and clustering of behavioural and neurodevelopmental disorders in DMD reveal the existence of a ‘DMD neuropsychiatric syndrome’ [13] which warrants prompt management and therapeutic intervention.

#### Animal Models

It is unlikely that animal models can fully recapitulate the DMD neuropsychiatric syndrome, although alterations in learning and behaviour are apparent. The most commonly used DMD disease model is the *mdx* mouse which harbours a naturally occurring nonsense mutation in *dmd* exon 23 resulting in an absence of full-length dystrophin expression [41]. Fear-motivated defensive behaviour, and according to some studies anxiety, are enhanced in *mdx* mice [42, 43]. Altered social behaviour and ultrasonic communication are noted [44] and learning and memory performance is impaired in some studies [45–49] but not others [50, 51]. Other *mdx* strains have been developed to explore the contribution of the different *DMD* gene products to pathology and disease phenotype. The *mdx3cv* mouse retains a low level of expression of near full-length dystrophin but is deficient in C-terminal dystrophin gene products [52]; Im et al. [53] show this to be the only *mdx* strain without Dp71 expression in the brain. The *mdx3cv* mice display enhanced anxiety-related behaviour and reduced locomotion in comparison with *mdx*, although surprisingly learning impairments are subtle and in some tests on par with *mdx* [54]. Dp71-null mice have been developed by homologous recombination; in contrast to *mdx* strains, they do not display muscular dystrophy [55]. These mice have deficits in cognitive flexibility, spatial learning and memory and cerebellum-dependent navigation strategies [56, 57]. Results from the Daoud et al.’s study [58] suggest that the learning deficits in Dp71-null mice are more severe than *mdx*. Reports of cognitive deficiency in any dog or large DMD animal models are scarce. In dystrophin-deficient miniature poodles, learning difficulties and abnormal behaviours have been reported, these dogs have a large deletion on the X chromosome encompassing the entire *DMD* gene [59].

### Gross Anatomical and Histological Observations

There is conflicting evidence relating to the presence and extent of anatomical brain abnormalities in DMD. Many studies report no gross central nervous system disturbances [60–63]. Other work describes both gross and histological abnormalities in DMD patients with varying degrees of severity [64–67]. Jagadha and Becker [66] report several neuropathological observations including neuronal loss, gliosis, Purkinje cell loss and abnormal dendritic development. One autopsy report of a DMD individual with severe intellectual disability revealed an atrophic brain with unusual multifocal small nodules in multiple layers of the prefrontal cortex [67]. The nodules were of astrocytic origin and believed to be a result of changes during early brain development. Other studies have also reported brain atrophy amongst DMD cohorts [68, 69]. Yoshioka et al. [68] observed slight cerebral atrophy in 67% of DMD patients and found that severity correlated with age. This is suggestive of a progressive cerebral degeneration, although there have been no longitudinal studies to confirm if the behavioural and cognitive symptoms themselves are progressive.

The head circumference of boys with Duchenne is larger than the normal population indicative of macrocephaly [70, 71]. Although the majority of DMD patients with relative, or absolute, macrocephaly are intellectually impaired, head circumference does not correlate with intellectual performance in DMD [70]. Interestingly, in Alzheimer’s disease patients with cerebral atrophy, a larger head circumference is associated with less cognitive impairment [72]. To some extent, the larger head circumference in DMD may be determined by hypertrophy of temporal muscles, skull shape is also significantly rounder in DMD patients compared with controls [73]. Conversely, no differences are found in skull morphology of *mdx* mice, presumably since bite force is unaffected in these mice [74, 75].

Electrophysiological studies using electroencephalograms (EEG) on DMD patients have been reviewed in 2002 [63], most studies report a higher proportion of abnormalities amongst DMD patient groups. A transcranial magnetic stimulation study showed reduced excitability of the motor cortex attributed to altered synaptic functioning and reduced dystrophin at the synapse [76].

An MRI study by Doorenweerd et al. [12] confirms the consensus that upon routine assessment, the brains of individuals with DMD (and animal models) show no gross abnormalities. Detailed quantitative analysis of images from an increased sample size did however reveal significantly smaller total brain and grey matter volumes in a DMD patient group. Further analysis showed significantly smaller grey matter regions in the left insula and occipital lobe, but these regions are too small to account for the reduced volume alone. White matter fractional anisotropy was also lower and radial diffusivity higher in DMD patients versus controls indicating that while white matter volume is unaffected, its structural organisation is compromised [12]. Smaller grey matter volume has been reported elsewhere [77] and Doorenweerd et al. suggest that, after correcting total brain volume to intracranial volume, altered brain maturation is responsible for smaller Duchenne brains rather than atrophy [12]. These alterations were more pronounced in patients lacking Dp140, patients lacking Dp71 were unavailable to this study. Independently of grey matter volume, cerebral blood flow (CBF) is lower (17%) in DMD patients compared with controls with no correlation to age, ambulation or cardiac involvement [12], CBF is also reduced to the same extent in the *mdx* mouse [78].

MRI studies in *mdx* mice have shown no significant differences in total brain volumes [75, 79, 80], although regional structural changes are apparent with reports of enlarged lateral ventricles [80], hippocampus, globus pallidus and caudate putamen and a smaller hypothalamus [75].

### Molecular Pathophysiology

Although shorter *DMD* transcripts are more abundant in the brain, full-length gene products are also produced (Table 2). Doorenweerd et al. [81] provide a detailed analysis of the spacial and temporal expression patterns of *DMD* transcripts in the healthy human brain. Expression from the dystrophin Purkinje promoter in mice produces Dp427p in cerebellar Purkinje cells, but such expression is absent in the human brain throughout development [81, 86] suggesting a different role for Dp427p in mouse than human and questioning the use of mouse models to study the brain in DMD. Interestingly, there is a cerebellar and hippocampal focus to biochemical alterations in DMD brains. Phonological dyslexia and verbal working memory deficits, commonly observed in DMD, are associated with the cerebellum [32, 40, 63, 93–95] and learning and memory are linked to the hippocampus. Cyrulnik in 2008 [21] argued that Duchenne is a cerebellar disorder, and recently in vivo, electrophysiology studies on *mdx* mice have revealed abnormal cerebellar circuit function [96]. The findings of Doreenweerd et al. in 2017 [81] emphasise the amygdala and hippocampus since it recently transpired that, in contrast to animal studies, *DMD* expression is highest in the hippocampus and amygdala and lowest in the cerebellum in the human brain.

**Table 2:** *DMD* gene products expressed in the brain

*DMD* product	Expression	Reference(s)
Dp427m	Low but detectable throughout brain development	[81–83]
Dp427c	Postsynaptic density of neurones in the cerebral cortex, hippocampus, amygdala and cerebellum	[81, 82, 84, 85]
Dp427p	Purkinje cells	[81, 86, 87]
Dp260	Retina	[88]
Dp140	Cerebral cortex during foetal development with some low expression postnatally in the cerebellum	[4, 81, 88]
Dp116	Schwann cells	[89]
Dp71	Ubiquitous and stable throughout development and adult life	[81, 90, 91]
Dp40	Ubiquitous	[92]

*m* muscle, *c* cortical, *p* Purkinje. Splice isoforms are not shown

In contrast to subtle changes in gross anatomy, abnormalities at the microscopic level are robust, with most knowledge arising from studies on the *mdx* mouse. The absolute number and the packing density of cells in the corticospinal system of *mdx* mice are 50% lower than controls and corticospinal neurones were found to have a rounder cell body than pyramidal controls [97]. There is also a significant reduction in hippocampal cell density [98].

Full-length dystrophin co-localises with GABA_A_ receptors in the mouse cerebellum, cerebral cortex and hippocampus [99]. GABA_A_ clusters are reduced in the *mdx* amygdala, cerebellum and hippocampus, most notably in cerebellar Purkinje cells, indicating a role for Dp427 in the clustering or stabilisation of GABA_A_ receptors in central inhibitory synapses [42, 99]. Similarly, the absence of Dp71 in other models is associated with altered clustering of the water channel aquaporin-4 and the Kir4.1 potassium channel in retinal glial cells as well as altered clustering and maturation of hippocampal neuronal glutamatergic receptors [58, 100]; these are discussed later in relation to the functional diversity of Dp71. Interestingly, in the *mdx* mouse brain, Dp71 is found in monomeric form compared with a crosslinker-induced oligomeric state observed in control brains [101].

Positron emission tomography (PET) studies have revealed regional glucose hypometabolism in DMD patients in dystrophin expressing regions. This appears unrelated to motor deficit and is indicative of lowered synaptic activity and is a common characteristic of disorders involving cognitive impairment [62, 102]. Altered glucose metabolism is also apparent in *mdx* mice which have significantly decreased free glucose levels and increased glucose use [103]. Like in dystrophic muscle, there are abnormal metabolite ratios in the brains of DMD patients and *mdx* mice such as a higher ratio of inorganic phosphate to ATP [104, 105]. These altered ratios do not correlate with cognitive profile or genotype and could be related to CO_2_ retention which is typically associated with neuromuscular disorders [63, 106]. Conversely, a proton magnetic resonance spectroscopy study by Doorenweerd reported a preserved biochemical composition in human DMD brains when compared with age-matched controls [107].

A chronically sustainable increase in choline-containing compounds has been reported in the frontal cortex and cerebellum of DMD patients and is not thought to be associated with intellectual disability but rather a beneficial compensatory mechanism [61, 103, 108]. In older *mdx* mice, the increase in choline-containing compounds is confined to the cerebellum and hippocampus [80, 103]. An elevation in choline-containing compounds is observed in a number of brain disorders and thought to be indicative of an unsustainable increase in membrane turnover from inflammation or increased cell division [61, 63]. The fact that in DMD, the increase is sustainable suggests that it is not due to changes in membrane turnover but is rather a permanent change in the level of water-soluble choline-containing compounds [61].

An age-related reduction in the water channel protein aquaporin-4 has been observed in astrocytic end feet surrounding capillaries in the brains of *mdx* mice. This was accompanied with swelling of the astrocytic perivascular processes which is an early indicator of brain oedema [109]. Altered cellular volumes have been reported in *mdx* brains [105] but no differences have been found in the response to hypoosmotic shock [103]. A reduction of Dp71 at glial end feet has been shown to alter blood brain barrier (BBB) development which is altered in the *mdx* mouse [110, 111]. Dystrophin expression parallels BBB development and perivascular glial arrangement in control mice [110, 112].

In *mdx* mice, CA1 hippocampal neurones have an increased susceptibility to hypoxia-induced reduction in synaptic transmission, this indicates a role for dystrophin in protecting neurones from hypoxia-induced damage [113]. Biochemical studies on learning and memory appear as conflicting as their behavioural counterparts described above, likely due to the nature of the training procedures used in memory and learning tasks. Sesay et al. [51] report no differences in long-term potentiation (LTP) in CA1 and dentate gyrus areas of the hippocampus in *mdx* mice compared with controls. Vaillend et al. [50] also reported no change in LTP in either *mdx* or the *mdx3cv* mouse which has altered expression of all dystrophin gene products. Instead, dystrophin deficiency appears to increase NMDA receptor–mediated short-term potentiation in *mdx* mice [50, 54, 114, 115], this may be due to the decreased GABA_A_ receptor clustering in dystrophin-deficient hippocampal neurones [114]. In a later study, using different training paradigms, Vaillend et al. did report enhanced CA1 hippocampal LTP in *mdx* mice demonstrating that memory defects may be corrected through specific training procedures, such as extended or distributed training [48]. Such altered synaptic plasticity may be responsible for the defects in memory consolidation in learning tasks observed in *mdx* mice: notably impaired long-term object recognition and impaired long-term spatial memory. An observed reduction in the number of CA1 pyramidal neurones in the anterodorsal hippocampus of *mdx* mice may also contribute to hippocampal-dependent learning and memory deficits in DMD [98].

In the Dp71-null mouse, the excitation/inhibition balance of the prefrontal cortex is shifted in favour of increased excitation. Whilst inhibitory transmission is unaffected, alterations of AMPA receptor–mediated glutamatergic transmission are apparent along with reduced synaptic plasticity [57]. Helleringer et al. in 2018 also report that Dp71 deficiency increases excitatory transmission [56]. They studied cerebellar physiology and function showing that enhanced transmission at climbing fibres on Purkinje neurones is linked to impairments in synaptic plasticity and the clustering of postsynaptic density protein 95 (PSD-95).

Collectively, both animal and human DMD studies have revealed molecular abnormalities centred around the hippocampus, amygdala and cerebellum. Given the predominance of Dp71 expression in these areas, and indeed the CNS as a whole, to understand the neuropathophysiology of Duchenne, a detailed understanding of this *DMD* gene product is essential.

## Dp71 Gene Expression and Splice Variants

**Fig. 2:**
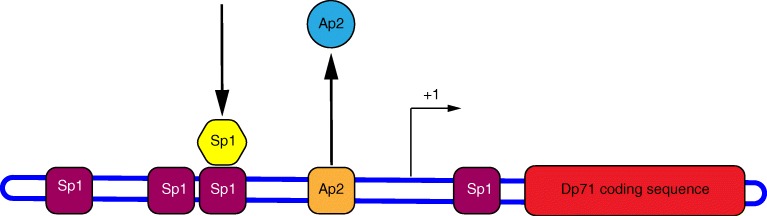
The Dp71 promoter region. Dp71 expression during neuronal differentitation is maintained by the combined action of Sp1 and AP2α as positive and negative regulators respectively. The transcription start site (+1) and Dp71 coding region are indicated

Dp71 (also known as apo-dystrophin-1) was first described as a 6.5-kb mRNA transcript in 1990 and subsequently confirmed to be encoded by a 4.8-kb mRNA present in all tissues with the exception of skeletal muscle [90, 91, 116–118]. Recently and contradictorily, Dp71 transcript and protein have been detected in skeletal muscle [119]. The promoter is approximately 8-kb upstream of Dp427m exon 63, the transcript has a novel first exon encoding a unique seven amino acid N-terminal sequence, MREQLKG [116, 120]. Thereafter, Dp71 shares exons 63–79 with full-length dystrophin which encode for the C-terminal and cysteine-rich domains. Dp71 therefore lacks the whole spectrin-like repeat and N-terminal actin-binding domains of full-length dystrophin, although the novel N-terminus of Dp71 does encode an actin-binding domain sufficient to localise Dp71 to actin filaments [121]. Studies on the promoter reveal it to be a housekeeping-type promoter as expected for ubiquitously expressed genes. It has a high GC content, four potential specificity protein 1 (Sp1)-binding sites and no TATA box [122]. Sp1 is a positive regulator and binds to GC-rich regions in the Dp71 promoter region to help define the transcription start site (Fig. 2). Dp71 is thought to be expressed during early myogenesis to aid in cytoskeletal remodelling [121] but its expression is then inactivated in mature muscle to prevent competition with full-length dystrophin for dystrophin-associated protein-binding sites [3, 123]. In contrast, in neurones, Dp71 expression remains strongly induced even in differentiated neurones. Sp1 works with activating protein 2α (AP2α) to maintain Dp71 expression, with AP2α acting as a negative regulator that is released from the AP2 motif during neuronal differentiation (Fig. 2) [124]. Dp71 expression has been shown to be developmentally regulated in several models. Transcript and protein are elevated during cAMP-induced differentiation of rat astrocytes [125] and nerve growth factor (NGF)–induced differentiation of PC12 cells [126, 127]. Sarig et al. [55] studied the differential activity of the Dp71 promoter during mouse development and showed that relatively high Dp71 promoter activity was observed alongside morphogenic events and terminal differentiation in several tissues including the CNS. Of note, the hippocampus showed particularly high activity in line with the latest findings by Doorenweerd [81]. The constitutive expression levels of Dp71 have been shown to be regulated post-translationally by the ubiquitin proteasome pathway in PC12 cells with phosphorylation being involved in the proteasome-dependent degradation of Dp71 [128].

Unlike muscle, the brain sees the highest regulatory complexity in RNA processing events. It is not surprising that the predominant dystrophin protein variants in the brain are alternatively spliced [129]. Several isoforms of Dp71 have been described as a result of alternative splicing. At the time of writing, we are aware of 14 Dp71 isoforms, although some are yet to be detected at the protein level (Fig. 1 and Table 3). This is in contrast to the recently identified Dp71 in skeletal muscle which is found with all exons intact [119]. Austin et al. [91] in 1995 were the first to characterise four canonical alternatively spliced Dp71 transcripts in cultured human aminocytes. The four isoforms differ according to the presence or absence of exons 71 and 78 (Fig. 1). The absence of exon 78 shifts the reading frame and creates a unique C-terminus by replacing the last 13 amino acids (RNTPGKPMREDTM) with 31 new residues (HNVGSLFHMADDLGRAMESLVSVMTDEEGAE). Dp71 lacking exon 71 was termed Dp71d and Dp71 lacking exon 78 termed the founder sequence, or Dp71f. Subsequently, this terminology was replaced with Dp71a and Dp71b respectively and the isoform missing both exons 71 and 78 termed Dp71ab. A fifth Dp71 isoform, first described in human foetal neural tissue, lacks exons 71–74 [130, 131]. The additional loss of exons 71–74 (an in-frame deletion of 330 bp) removes the 110 amino acid syntrophin–binding domain and the isoform is detected as a 58-kDa protein on western blot. The isoform, termed Dp71_Δ110_, can also be alternatively spliced for exon 78 creating a sixth isoform [131]. To our knowledge, Dp71_Δ110_ has only been detected in the CNS where it represents a relatively small proportion of Dp71 isoforms in the brain [131]. In 2012, Saint Martin et al. [132] described a new alternative splicing event in rat PC12 cells that gives rise to the Dp71e isoform. Dp71e retains the last 34 bp of intron 77 that results in a frameshift and premature stop codon preventing the translation of exons 78 and 79. The unique C-terminus of Dp71e is hydrophilic and composed of 10 amino acids, DLSASSSLYY. Subsequently, two Dp71e isoforms with exon 71 or exons 71–74 removed have been described in rat PC12 cells [133]. Aragon et al. [133] have further extended the repertoire of Dp71 isoforms using mouse brain and retina tissue to identify four new isoforms with varying splicing patterns between exons 71 and 74 (Fig. 1). Finally, the most recently discovered isoform of Dp71 is deleted for exons 68–76 and exon 78 (the smallest yet, predicted to be 25 kDa) and was described in a glioblastoma cell line and termed Dp71b_Δ68-76_. Dp71b_Δ68-76_ has a unique C-terminus due to a stop codon in exon 77, the unique amino acids are VRKIFSVLPRTQAQG. These authors also describe an isoform missing exons 71, 73 and 78 termed Dp71ab_△73_ [134]. Rani report such Dp71′b′ type isoforms to be the major type of Dp71 in glioblastoma cell lines. Aragon et al. [135] have demonstrated that the Dp71d group of isoforms is highly expressed in the brain, while the Dp71f group predominates in the retina; Dp71e group is thought to be expressed at very low levels [135].

**Table 3:**
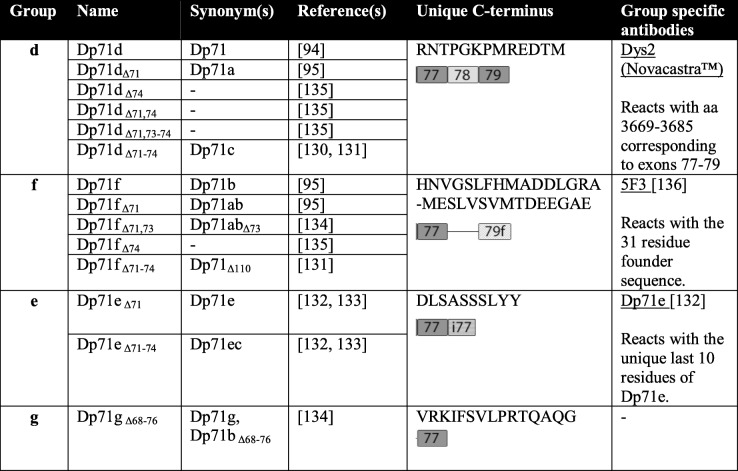
Dp71 isoform grouping and nomenclature

Dp71 isoforms have a complicated nomenclature. Recently, the multitude of additional splice variants has prompted their grouping according to C-terminal structure. Current, and favoured, nomenclature was described by Aragon et al. in 2018 [135] and is updated here in Fig. 1 and Table 3. In line with this favoured nomenclature and as proposed by Rani et al. [134], the Dp71b_Δ68-76_ isoform forms a new group, g, and can be renamed Dp71g_Δ68-76_. The d group of Dp71 isoforms contain exons 78 and 79, the f group lacks exon 78 and has an alternative exon 79 (79f) and the e group contains part of intron 77 (i77) and lacks exons 78 and 79. The f group isoforms have a more hydrophobic C-terminus than the d and e groups and can be detected with specific antibodies such as 5F3 [3, 136]. The g group isoform has a stop codon in exon 77.

Although some isoforms have yet to be detected at the protein level, it is clear that RNA processing is responsible for the ubiquity and functional diversity of Dp71 in the brain.

## Functional Diversity of Dp71

The multitude of Dp71 isoforms described above makes functional studies difficult. Many studies and reviews concerning the DMD neuropsychiatric syndrome have considered Dp71 a single protein and many use a pan Dp71 antibody unable to discriminate between splice isoforms. It is important that the repertoire of isoforms in a chosen model be established alongside any functional studies. This has been done for example in a glioblastoma cell line [134] and PC12 cells [133], the latter being the most common cellular model to asses Dp71 function. PC12 cells are derived from a pheochromocytoma of the rat adrenal medulla. The tissue has an embryonic origin from the neural crest with populations of neuroblastic cells. It must be noted that this model system has some limitations for the study of the human nervous system. In this review, the term Dp71 should be taken to encompass the whole family of isoforms unless otherwise stated; where specific splice isoform data is available, we use current nomenclature described above.

**Fig. 3:**
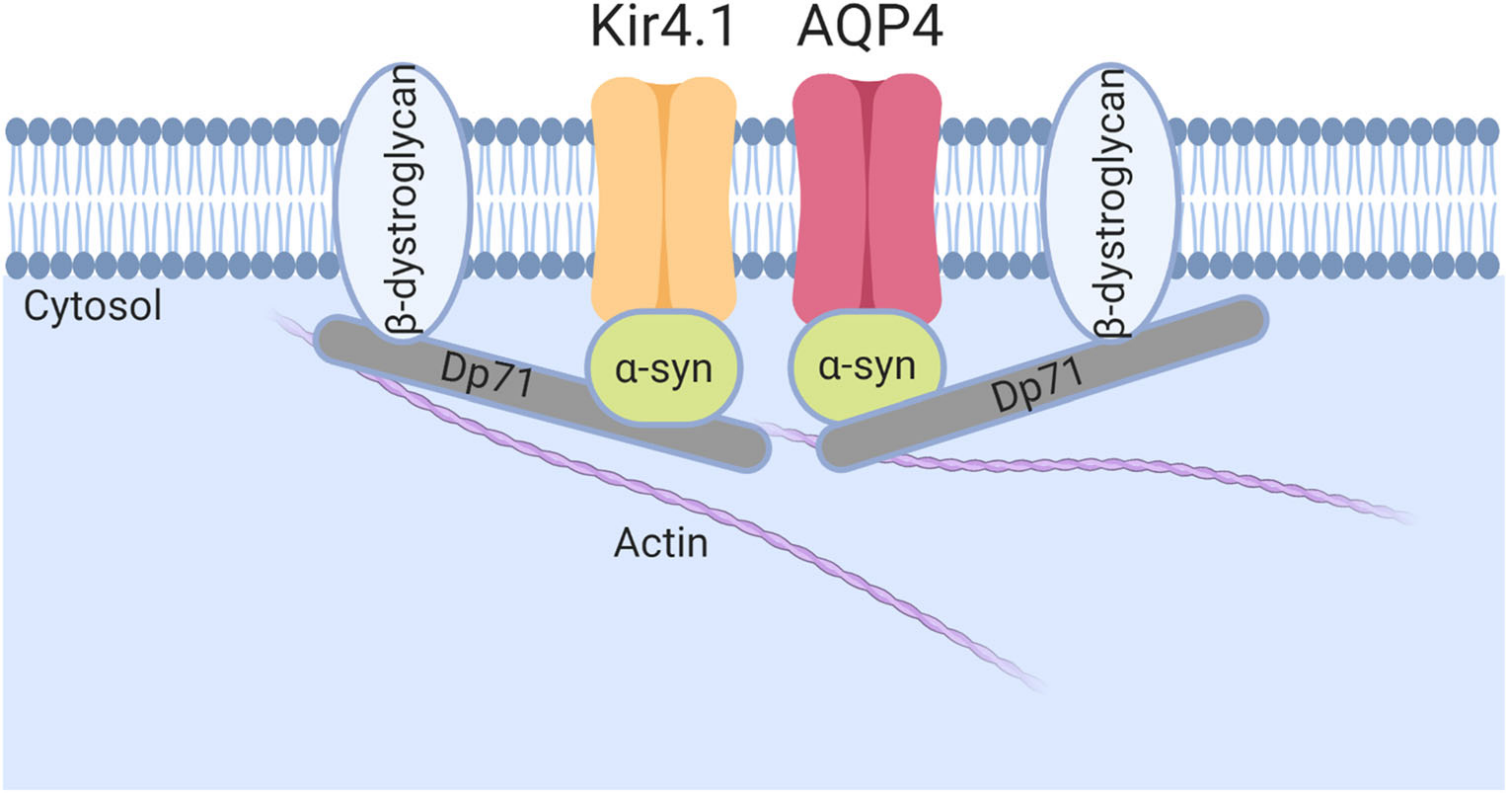
The canonical non-neuronal role of Dp71. In the retina, Dp71 anchors aquaporin 4 (AQP4) and the inwardly rectifying potassium channel (Kir4.1) at the glial perivascular end-feet of Müller glial cells. α-syn, alpha-syntrophin

**Fig. 4:**
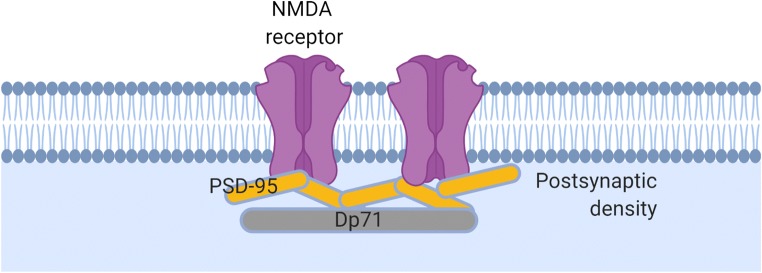
A proposed synaptic function for Dp71. Dp71 organises glutamate receptor distribution at the postsynaptic density. The exact position and binding of Dp71 in relation to PSD-95 and particular receptors is unclear

A canonical function of Dp71 in the brain relates to the clustering of the water channel aquaporin-4 (AQP4) and the inwardly rectifying potassium channel Kir4.1 in retinal Müller glial cells [137, 138]. It is well documented that DMD patients with mutations towards the centre and 3′ end of the *DMD* gene have an abnormal electroretinogram. Retinal Müller glial cells are responsible for maintaining retinal homeostasis; Dp71 is the only dystrophin gene product expressed in these cells and it anchors AQP4 and Kir4.1 channels at the glial perivascular end-feet which are essential for osmoregulation and potassium buffering respectively (Fig. 3) [139, 140]. Retinal Müller glial cells are a common cell model used to study Dp71 [3] and a clear role for Dp71 in retinal osmoregulation and vascular permeability of the retina is established. A role for Dp71 in the clustering of ion channels and receptors is not limited to glial cells with Dp71 reported to play a role in the clustering and maturation of glutamatergic receptors in hippocampal neurones (Fig. 4) [58]. Dp71-null mice have increased excitatory transmission, aberrant synapse density, organisation and maturation as well as reduced synaptic plasticity in the CA1 hippocampus [58, 100]. Beyond these roles in glia, synapse functions and neuronal excitability, the emerging complexity of Dp71 splice isoforms and their differential subcellular localisations has led to an expanding repertoire of functions which we discuss below.

Dp71 has long been confirmed a cytoskeletal plasma membrane–associated protein in various tissues and cells including cultured rat brain astrocytes [3]. Like full-length dystrophin in muscle, Dp71 forms a dystrophin-associated protein complex (DAPC) in the brain [3]. Dp71 therefore retains some recognisable functionality of full-length dystrophin through binding with dystroglycans, dystrobrevins and syntrophins since their binding domains are located near the C-terminus. The dystroglycan-binding site on dystrophin has been delineated to amino acids 3054–3271 (spanning exons 61–68, [141]) whilst syntrophin-binding sites are coded by exons 73–74 [142]. Although Dp71 is able to restore the DAPC in skeletal muscle, it does not compensate for the lost function of Dp427; thus, even though Dp71 interacts with some of the same proteins, it likely has different functions than full-length dystrophin [91]. The existence of Dp71 splice isoforms without the capacity to bind such dystrophin-associated proteins demonstrates a complex functional diversity regulated via RNA processing. Blake et al. [143] indicate differences in DAPC composition between neurones and glia and show Dp71 to be associated with α-dystrobrevin-1 and syntrophin in glia; interestingly, such complexes are still formed in *mdx3cv* mice which lack C-terminal dystrophins, although later studies have shown disruption of such complexes in these mice [144]. Dp71-DAPCs in several tissues have since been characterised and revealed to differ according to cell type and microenvironment (reviewed by [3]). Dp71-DAPCs in the CNS contain the following: β-dystroglycan, δ-sarcoglycan, α1-syntrophin in rat retinal Müller glial cells [145] and α-dystrobrevin-1, α1- and β2-syntrophin, and α- and β-dystroglycan in glial end-feet and vascular endothelial cells [144]. In undifferentiated PC12 cells, Dp71f_Δ71_ is associated with β-dystroglycan, β1-syntrophin, β-dystrobrevin and α-, β-, and γ-sarcoglycan [146]. During NGF-induced differentiation, the complex is altered to contain β-dystroglycan, α1-syntrophin, β-dystrobrevin, γ-sarcoglycan and neuronal nitric oxide synthase (nNOS). The latter hints at an involvement for Dp71f_Δ71_ in signal transduction during neuronal differentiation [146].

A role for Dp71 in neuronal differentiation is supported by the fact that Dp71d and f are upregulated during PC12 cell differentiation. Reduced Dp71 expression inhibits NGF-induced neurite outgrowth and neuronal differentiation in PC12 cells [147]. Acosta et al. show that the late differentiation marker, microtubule-associated protein 2 (MAP2) is inhibited along with Dp71 and that the loss of Dp71 also correlates with altered dystrophin-associated protein expression. Furthermore, Aragon et al. 2016 [133] identified Dp71 isoforms in PC12 cells localised to neurite extensions and growth cones suggesting a role in differentiation and neurite growth, also supported by [58] who show Dp71f expression in the growth cones of cultured neurones from Dp71-null mice, more so in excitatory synapses. The splice isoform Dp71e_Δ71_ has also recently been shown to have a role in neurite outgrowth in PC12 cells through regulating the expression of the cytoskeletal proteins HspB1, S110A6 and K8 as well as the HCNP protein involved in neurotransmitter synthesis [148]. These authors overexpressed Dp71e_Δ71_ during neuronal differentiation and found overexpression to increase neuronal differentiation and alter the expression profile. Cytoskeletal reorganisation and neurotransmitter synthesis are both required for differentiation which appears a key role for Dp71 in neurones. In support of these findings, the same group generated a Dp71 mutant lacking exons 78 and 79, Dp71_Δ78-79_ which they show to stimulate PC12 proliferation [149], cell differentiation [150] and neurite outgrowth through the phosphorylation of HspB1 [151].

**Fig. 5:**
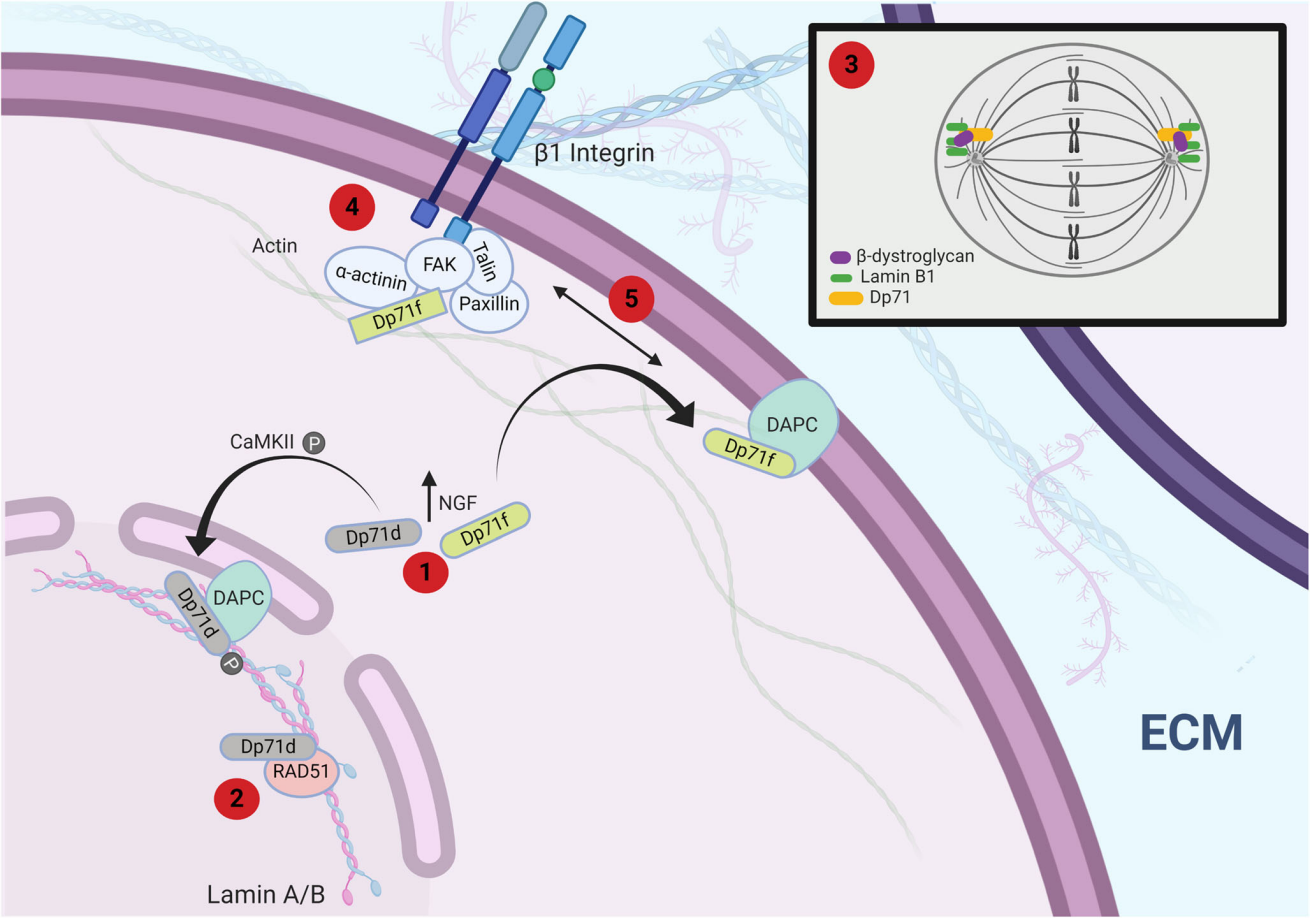
Multiple non-synaptic roles of Dp71. (1) Upon NGF-induced differentiation, Dp71d is phosphorylated by CaMKII and relocates to the nucleus where it forms a nuclear DAPC and may mediate nuclear responses during NGF signalling. In contrast, Dp71f moves to the cell surface and localises with the DAPC in areas such as the growth cone to play a role in neurite outgrowth. (2) Nuclear Dp71d binds to the DNA repair protein, RAD51. It also plays a role during cell division (3). (4) Dp71f is a facilitator of cell adhesion and binds to the β1-integrin adhesion complex. (5) Dp71 aids in cross-communication between the DAPC and the β1-integrin adhesion complex. Figure created with Biorender.com

Dp71 expression is not confined to the plasma membrane indicating it has different functions within the same cell (Fig. 5). Both Dp71d and Dp71f have been found in cultured neuronal nuclei [152]. Using Dp71 group–specific antibodies, Dp71d was found within nuclear granules in both neurones and astrocytes whilst Dp71f nuclear staining was only observed in neurones [152]. Dp71f and Dp71f_Δ71_ are also localised to the nucleus of HEK293 cells with Dp71f appearing more dominant and Dp71f_Δ71_ specific to only the nucleus [153]. Alternative splicing determines the subcellular localisation of Dp71. Using GFP-tagged constructs with or without exons 71 and 78, Gonzalez et al. [154] determined that the construct lacking both exons 71 and 78 (Dp71f_Δ71_) is found exclusively in the cytoplasm of HeLa, C2C12 and N1E-115 cells whilst Dp71d_Δ71_ was found exclusively in the nucleus. Dp71d and Dp71f, containing exon 71, had both nuclear and cytoplasmic localisations. Marquez in 2003 [127] later confirmed the exclusive localisation of Dp71d_Δ71_ and Dp71f _Δ71_ to the nucleus and cytoplasm of PC12 cells respectively. The presence of either exon 71 or exon 78 therefore appears to determine the subcellular localisation of Dp71. Furthermore, during NGF-induced PC12 cell differentiation, Dp71f _Δ71_ relocates from the cytoplasm to neuritic processes such as the growth cone and Dp71d_Δ71_ appears to relocate almost entirely to the nucleus where it binds the nuclear matrix during the late stages of neuronal differentiation in PC12 cells (Fig. 5) [127, 155], thus not only are Dp71 levels upregulated but there is a differential subcellular localisation of Dp71 isoforms during neuronal differentiation. Garcia Cruz et al. [148] also document an increase in nuclear expression of Dp71e_Δ71_ during neuronal differentiation. Interestingly, throughout the process of differentiation, Dp71 maintains a co-localisation with β-dystroglycan, including during the relocation of Dp71d_Δ71_ to the nucleus where levels of β-dystroglycan are also increased [127]. Dp71 is known to interact directly with β-dystroglycan, the most abundant dystrophin-associated protein that binds intracellularly to dystrophin and extracellularly to α-dystroglycan forming a strengthening link between the extra cellular matrix and the cytoplasm [156]. Thus, it is suggested that a Dp71-containing DAPC exists in the nucleus of neuronal cells. Indeed, Dp71 co-immunoprecipitates with sarcoglycans, β-dystroglycan, syntrophins and dystrobrevins in HeLa, C2C12 and PC12 nuclear fractions where a nuclear DAPC is formed [3, 157–159].

Dp71 does not contain a nuclear localisation signal (NLS) but, like other nuclear proteins, phosphorylation appears to regulate its nuclear transport. Dp71d_Δ71_ is phosphorylated at serine and threonine residues in PC12 cells by protein kinase C (PKC) and the Ca2+/calmodulin-dependent protein kinase II (CaMKII) with NGF treatment stimulating phosphorylation and CaMKII-phosphorylated Dp71d favouring a nuclear localisation (Fig. 5) [155]. It is suggested that a CaMKII phosphorylation site located in exon 79 modulates the nuclear localisation of Dp71d_Δ71_ since this site is not present in the unique C-terminus of Dp71f group isoforms. Phosphorylation of dystrophin is known to mediate its interactions with members of the DAPC and actin [155] and β-dystroglycan contains a NLS [160]; therefore, Dp71 may access the nucleus through its interaction with β-dystroglycan [3]. More recently, it was determined using truncation mutants in C2C12 cells that Dp71d shuttles between the nucleus and cytoplasm through association with importin (IMP) α/β and the exportin CRM1 [161]. These authors found the cysteine-rich zinc-finger ZZ domain is required for import and that Dp71 likely utilises microtubules and the motor protein dynein for retrograde transport to the nucleus from the plasma membrane [161]. The alternative splicing of Dp71 may control phosphorylation events (and ultimately function) since phosphorylation of Dp71 by cyclin-dependent kinase 1 (CDK1) occurs at a site in exon 78; such a site is not present in utrophin, a protein with high homology to dystrophin [91]. Thus, Dp71 is precisely and differentially regulated during PC12 cell differentiation.

Besides the plasma membrane and nucleus, Dp71d and Dp71f group isoforms have been observed in the Golgi apparatus and Dp71d in the neurofilament cytoskeleton [152]. Dp71d group isoforms are also reported to be localised in microsomes and a Dp71f-like protein is expressed in mitochondria of *mdx3cv* mice [162].

Taken together, RNA processing is important for the correct localisation and function of dystrophin in the brain with the C-terminal end in particular determining the location and thus function of each isoform.

### Adhesion

Given Dp71 isoforms are differentially expressed and localised during neuronal differentiation and the apparent role of Dp71 in signalling during neuronal differentiation, it is unsurprising a role for Dp71 in adhesion has been described given that adhesion and differentiation are closely linked.

The DAPC is known to interact with the integrin adhesion system including proteins such as the integrins, viniculin, talin, paxillin and focal adhesion kinase (FAK); evidence now links Dp71 with cell adhesion in the brain [3]. In the glioblastoma cell line, U-373 MG Garcia-Tovar et al. showed a Dp71f group isoform localised to the focal complex and leading borders of lamellipodia where it co-localises with vinculin, β1-integrin and α-actinin [163]. Dp71f_Δ71-74_ and Dp71d_Δ71-74_ also appear to be involved in the remodelling of the platelet cytoskeleton where in *mdx3cv* mice thrombin-mediated platelet adhesion is impaired [3, 164]. Dp71f_Δ71_ binds to the adhesion complex in PC12 cells, associating with FAK, β1-integrin, actin, talin and α-actinin (Fig. 5) [165, 166]. The depletion of Dp71 results in a reduction of β1-integrin adhesion complex components and deficient adhesion to laminin [166]. Cerna et al. further demonstrate that Dp71f_Δ71_ binds directly to FAK and β1-integrin, thus Dp71 is a structural component of the β1-integrin adhesion complex in PC12 cells required for correct function and/or assembly and stability [166]. Interestingly, in the study by Cerna, β-dystroglycan was not detected in immunoprecipitates of β1-integrin and FAK suggesting the binding of β-dystroglycan to Dp71 is out of the context of the adhesion complex.

Adhesion, neurite outgrowth and differentiation are closely linked, it is proposed that the deficient adhesion activity of Dp71f_Δ71_-depleted PC12 cells is responsible for their lack of NGF-induced neurite outgrowth [3, 147]. Cerna et al. proposed a model which we update in Fig. 5 of how Dp71f_Δ71_ associates with the β1-integrin adhesion complex. Its association with α-actinin suggests crosstalk between the Dp71-containing DAPC and the adhesion complex with Dp71 conferring stability [166]. Such intricate functions require investigation in DMD and human-derived model systems.

### Cell Division

Dp71 depletion studies in PC12 cells have revealed a role in cell division. Dp71-depleted cells have reduced growth with a delay in G0/G1 transition and an increase in apoptosis during nocodazole-induced mitotic arrest [167]. Dp71 localises with lamin B1 and β-dystroglycan (which are involved in cell division, [168, 169]) at the mitotic spindle poles, cleavage furrow and midbody, these proteins are reduced at these sites upon Dp71 depletion in PC12 cells [167]. Dp71 is therefore considered a component of the mitotic spindle and cytokinesis protein complexes and could modulate the cell division cycle through interactions with lamin B1 and β-dystroglycan (Fig. 5). Based on the antibodies used and knowledge of Dp71 isoform expression in PC12 cells, the Dp71d_Δ71_ splice variant with predominant nuclear localisation and an intact exon 78 is implicated here.

Of relevance to a role in cell division, it is interesting to note the poorly defined, but intriguing, role of dystrophin, and Dp71 in particular, in tumourigenesis where its association with proteins such as FAK implicate Dp71 in cell migration and invasion [170, 171]. This has been reviewed by [172] and is outside the scope of this review.

### Nuclear Function(s)

The proposed role of Dp71 in the nucleus is primarily as a scaffolding protein at the nuclear envelope [3]. This is supported by the presence of nuclear Dp71d group containing DAPC complexes and the presence of Dp71d in the nuclear matrix and/or envelope in various cell lines as discussed above (Fig. 5). Dp71 also directly interacts with the nuclear envelope protein lamin B1 [157, 158]. Such a nuclear scaffolding role implicates Dp71, indirectly, in nuclei morphology, gene expression and DNA repair since these functions, and others, utilise the support of the nuclear matrix and/or envelope. The nuclear envelope consists of two lipid bilayer membranes and nuclear pore complexes. Lamins are intermediate filaments associated with the inner nuclear membrane and are the principle component of the nuclear matrix. Nuclear matrix–associated Dp71d_Δ71_ increases during neuronal NGF-induced differentiation and relocates from the periphery of the nuclear matrix to the centre of the nucleoskeletal structure [173]. The depletion of Dp71 results in reduced levels of lamin B1 and the mislocalisation of emerin, an integral protein of the inner nuclear membrane [167, 173]. Such changes to Dp71d_Δ71_ localisation and expression demonstrate the dynamic nature of Dp71d_Δ71_ in the nucleus and further evidences the role of Dp71 during neuronal differentiation. To further support an extended role for Dp71 in nuclear functions, Dp71 has been shown through immunoprecipitation and immunocytochemistry to bind to RAD51 in human bronchial epithelial cells [174]. These authors do not determine the specific isoform(s) of Dp71 in their study but depletion of Dp71 increases DNA damage and H_2_O_2_-induced apoptosis. RAD51 is a protein involved in homologous recombination repair upon DNA damage; it additionally binds to lamin B1 (Fig. 5) and its binding to Dp71 in epithelial cells warrants further investigation in neuronal cell models as does the overall nuclear function of Dp71 in DMD and human-derived model systems.

Dp71 functions are diverse but they include closely related processes such as differentiation, neurite outgrowth and cell adhesion. It would be interesting for future studies to delineate the Dp71-binding sites and isoform specificity of protein-protein interactions in relation to the functions described above. Utrophin is a foetal homologue of dystrophin and is regarded as a potential replacement for dystrophin loss in DMD. Interestingly, utrophin upregulation in the brain does not ameliorate the behavioural deficits in *mdx* mice [175] highlighting the functional importance of specific *DMD* gene products such as Dp71 in the brain and their association to the neuropathophysiology of the disease.

## Role of Dp71 in the Neuropathophysiology of DMD

### Genotype-Phenotype Correlations

The existence of ‘hot-spots’ for *DMD* gene mutations, and exceptions to the reading frame hypothesis [2], has instigated many DMD and Becker muscular dystrophy (BMD) genotype-phenotype relationship studies. BMD is a milder condition caused by in-frame mutations in the *DMD* gene, BMD patients express varying levels of dystrophin protein. Enhanced genetic testing and genotype definition in the dystrophinopathies has led to a growing understanding of the genotype-phenotype relationship that is proving beneficial for diagnostic and treatment purposes [2, 176, 177].

Original studies in the 1990s showed no genotype-phenotype correlation in relation to the DMD neuropsychiatric syndrome, but these were conducted with limited understanding of C-terminal dystrophin gene products and the complexity of the *DMD* gene [2, 178, 179]. It is now clear that the frequency and severity of CNS involvement increase with the accumulated loss of distal dystrophin gene products, with *DMD* mutations after exon 63 (and therefore Dp71 loss) associated with the most severe intellectual impairment [2, 7–9, 11, 180–182]. Mutations located after exon 63 are rare and affect the expression of Dp71, as well as all other brain *DMD* gene products. All individuals with mutations disrupting Dp71 expression reported to date have severe intellectual impairment, it has even been known for brain comorbidities such as autism to precede a diagnosis of Duchenne [2]. Desguerre et al. in 2009 identified four sub-populations of DMD based on the severity of muscle and brain dysfunction; they show that mutations upstream of exon 30 correlated well with spared cognition but not motor function [183]. Most recently, it is reported that, in steroid-naïve boys with dystrophinopathy, speech delay and learning difficulties are more common in boys with mutations downstream of *DMD* exon 45 [39]. In 1998, Moizard et al. reported two patients with severe intellectual disability with *DMD* mutations altering Dp71 transcripts [7]. The same group later demonstrated amongst a larger cohort without *DMD* deletions or duplications who have severe intellectual impairment that all patients with point mutations resulting in the termination of Dp71 expression were the most severely affected [8]. A large study on 81 DMD and BMD patients with mutations either affecting all dystrophin gene products (54 patients) or all products except Dp71 (27 patients) found that the BMD patients with intellectual disability had mutations affecting Dp71 expression and that mutations upstream of exon 62 in DMD patients are associated with normal to borderline cognitive performance [6]. Ricotti et al. add further support in an even larger multicentre study of 130 DMD patients reporting a higher incidence of intellectual disability in 14 patients with distal *DMD* mutations affecting all gene products [13]. They report 64% of individuals with mutations affecting Dp71 had intellectual disability compared with 25% of those with mutations affecting Dp260, Dp140 and Dp116, whilst only 15% of patients presented with intellectual disability when the mutations only affected full-length dystrophin [13]. The group with Dp71 disrupting mutations also had the most severely affected working memory and highest Social Communication Disorder Checklist (SCDC) score.

### Tissue and Cellular Evidence

The localisation and function of Dp71 variants in the brain combined with the genotype-phenotype observations described above provide strong support for the involvement of Dp71 in cognitive impairment and the DMD neuropsychiatric syndrome. There are conflicting reports concerning the level to which Dp71 expression is affected in *mdx* mice. Nico et al. show reduced levels of Dp71 in the brains of *mdx* mice compared with controls indicating that *mdx* mice may be a model of Dp71 deficiency [184]. However, this is contradicted in other studies including in our own unpublished data. An important model for understanding how Dp71 loss can contribute to cognitive impairment is the Dp71-null mouse. Daoud et al. in 2009 carried out the first detailed functional and behavioural characterisation of the brain in these mice [58]. Cultured primary neurones had abnormal glutamatergic synapse maturation and organisation and altered synapse density [58]. Glutamatergic transmission was enhanced and synaptic plasticity reduced in the CA1 hippocampus. These mice have a reduced exploratory and novelty-seeking behaviour as well as deficits in spatial learning and memory [58]. Dp71-null mice display altered expression of some, but not all, DAPC proteins, for example β-dystroglycan and α-syntrophin expressions are reduced whilst γ1-syntrophin is overexpressed by more than 200%, α-dystrobrevins were unchanged indicating a requirement of Dp71 for the anchoring of some but not all DAPC proteins [58]. Dp71 loss in cultured neurones from these mice affected the clustering and distribution of the synaptic proteins type-1 vesicular glutamate transporter (VGLUT1) and PSD-95; Dp71 loss is believed to increase the stabilisation and/or local translation of PSD-95 [56, 58]. The mice have abnormally large clusters of PSD-95 and a reduced number of excitatory synapses. Dp71 therefore may organise glutamate receptor distribution (Fig. 4). Dp71-null mice have reduced synapse density and the morphology of the postsynaptic active zone is disrupted. Daoud et al. suggest that a postsynaptic mechanism contributes to the enhanced synaptic transmission in Dp71-null mice [58]. The unusual accumulation of PSD-95 in Dp71-null synapses could increase NMDAr gating and delivery to the synapse thus increasing AMPAr currents. Thus the role of Dp71 is not directly in synaptic plasticity but rather organisation and maturation of the synapse.

The morphogenesis and plasticity of the synapse are known to play a role in intellectual disability [185] and thus it can be speculated that the lower IQ levels observed in DMD patients may be explained by altered synapse organisation and maturation. The behavioural characteristics of the Dp71-null mouse are also in agreement. These mice display reduced exploratory behaviour, slight retention deficits in inhibitory avoidance and impaired spatial learning and memory [58]. Deficits in exploratory behaviour have been linked to enhanced glutamatergic transmission [186], although this behaviour to our knowledge has not been specifically assessed in DMD patients although could be postulated from the clinical observations discussed earlier. Dp71-null mice have more severe learning impairments than *mdx* mice supporting the view that Dp71 plays a role in the neuropathophysiology of DMD. The respective and combined involvement of both GABAergic and glutamatergic functions in the basis of intellectual disability in DMD remains to be elucidated; since unlike Dp71-null mice, in DMD patients, a cumulative loss of Dp427, Dp140 and Dp71 is likely a key contributor to the severity of brain involvement in DMD. Further studies on the *mdx3cv* mouse model may shed light here, although compensatory mechanisms such as from the small level of Dp427 present add a complication.

Induced pluripotent stem cells (iPSCs) prepared from patient-derived blood or fibroblasts are fast becoming an important tool for studying the disease pathogenesis and represent a human model to study the neuropathogenesis of DMD. The latest technology has seen the differentitation of skeletal muscle cells that recapitulate key DMD disease features [187]. In 2019, two papers described alterations in iPSC-derived neurones and astrocytes from DMD patients with variable deletions. Patel et al. characterised five DMD astrocytic lines from patients with deletions affecting the expression of either Dp427, Dp427 and Dp260 or Dp427, Dp260 and Dp140 [188]. Behavioural problems or autism is described in three patients, all of which have mutations affecting all three *DMD* gene products. Interestingly, cortical neuronal progeny from these patients did not show any obvious abnormalities consistent with the fact that neurones do not express Dp427 in sufficient amounts and Dp71 remains unaffected in these lines. Cytoskeletal abnormalities (increased cell area, volume and branching), alterations in Ca^2+^ handling and nitric oxide signalling were observed in iPSC-derived astrocytes from DMD patients when compared with controls. Patel demonstrate that iPSC-derived astrocytes from DMD patients have significant defects in glutamate handling with decreased glutamate uptake/consumption, they find that increased new production of glutamate by these astrocytes in turn causes neuronal defects such as decreased neurite outgrowth and hyperexcitability and reactive astrogliosis. It is interesting that Dp71 is preserved in these astrocytes and studies using iPSC-derived from DMD patients with mutations affecting Dp71 are warranted. Ruggieri et al. in 2019 report on iPSC-derived neurones (glutamatergic sensory lineages) from a DMD patient with a mutation in intron 70 and reduced Dp71 expression in the derived cells [189]. The patient has mid-level intellectual disability. iPSC-derived neurones from this patient were smaller in both perimeter and surface area and have irregular nuclei compared with control; adult neural stem cells from *mdx* mice are also reduced in size [111]. Using the pan-Dp71 antibody MANDRA1, Dp71 was not strongly associated with the membrane and scattered in the cytoplasm in contrast to the control. DMD-differentiated neurones were able to produce an action potential but were more typically unipolar or bipolar compared with the multipolar controls, DMD neurones presented with more strongly spread out processes than controls. Ultra-structurally, the patient neurones had dilated processes containing autophagic vacuoles. Ruggieri et al. report an increase in the expression of the sarco/endoplasmic reticulum Ca^2+^-ATPase (SERCA2) pump in iPSC-derived neurones from the DMD patient as well as cytosolic Ca^2+^ overload. SERCA2 is located on the endoplasmic reticulum (ER) membrane and is a key protein responsible for clearing intracellular Ca^2+^. SERCA2 dysregulation is implicated in disorders affecting cognitive ability [190]. Thus, Ca^2+^ dyshomeostasis occurs in Dp71-reduced sensory neurones as well as in skeletal muscle. An increase in the release of Ca^2+^ from intracellular stores (SR/ER) is associated with spatial learning deficit in *mdx* mice since a reduction of [Ca^2+^]i improves cognitive function in *mdx* mice [191]. This is the first study from a human model demonstrating abnormalities in Dp71-deficient neurones that are associated with cognitive impairment. Further studies on iPSC-derived neuronal and glial cells from Dp-71-deficient DMD patients are required to elucidate the precise cellular processes and mechanisms that may contribute to the neuropathogenesis of DMD.

Using transcriptomic data from Allen Human Brain and BrainSpan atlases, co-expression analysis of Dp427, Dp170 and Dp71 + Dp40 has revealed an association with genes involved in neurodevelopmental disorders [81]. Genes co-expressed with Dp71 + Dp40 (undifferentiated in this study) were enriched in gene ontology terms related to wound healing, cell motility, actin cytoskeleton and receptor binding. The top phenotype identified was abnormal cerebral vasculature. This Doorenweerd et al. study implies that the structural abnormalities identified in the brains of patients lacking Dp427 and Dp140 are aggravated further in patients also missing Dp71 and Dp40. Several X-linked developmental disorders linked to intellectual impairment are associated with proteins involved in regulating cytoskeletal organisation at excitatory synapses [185]. Further, abnormal neuronal migration during development is associated with autism spectrum disorders [192]. Given Dp71 binds to key regulators of this process such as FAK and β1-integrin, we investigated a role for Dp71 in cell migration finding that DMD patient–derived fibroblast cell lines lacking Dp71 migrate at a faster rate than control [170]. β1-integrin-meditated cell adhesion modulates neuronal migration and synaptogenesis during central nervous system development [193]. It is reasonable to suggest that neuronal migration be disturbed in DMD patients lacking Dp71. In support of this, work by Niks et al. [194] suggests that the changes in the DMD brain are structural and indicate perturbed brain development as opposed to progressive cerebral damage. Indeed, cerebral heterotopia, where neurones do not migrate properly, has been recorded upon autopsy in 1988 where it was also suggested abnormal dendritic development and branching may underlie intellectual impairment [66]. The increased excitation in prefrontal networks identified in the Dp71-null mouse affects executive functions critical for intellectual function in humans [57]. In line with alterations in neurodevelopment, excitation/inhibition imbalance along with executive dysfunctions is associated with the pathogenesis of neurodevelopmental disorders comorbid for intellectual disability and neuropsychiatric disorders [57].

Glial cells have an important physiological role in regulating neuronal excitability through the buffering of potassium; abnormal potassium clearance is linked to epilepsy [195]. The incidence of epilepsy in DMD patients can convincingly be tied to dystrophin expression and the absence of Dp71 in particular. This has been reviewed in [196]. For example, Dp71 is preferentially expressed in the amygdala and hippocampus, two areas associated with epileptogenesis and the functions of Dp71 in the clustering of potassium channels and maintaining BBB integrity are also strongly linked to epilepsy.

An altered prevalence of AQP4 and Dp71 is observed in the neurodegenerative disease idiopathic normal pressure hydrocephalus (iNPH) and their dysfunction is associated with slowly evolving neurodegeneration in this condition [197]. Neurodegeneration as a concept has not been explored in Duchenne since there is a presumption that the neuropsychiatric phenotype as a whole is not progressive. Pairing recent insights into the function and involvement of Dp71 in the neuropathophysiology of DMD with original autopsy findings of cerebral heterotopia, neuronal loss, gliosis and Purkinje cell loss [66] is worth exploring.

## Conclusion

The high risk of neuropsychiatric syndromes in DMD patients warrants early intervention to achieve the best possible quality of life. We have entered an unprecedented era in DMD research with new drugs entering the market that can restore dystrophin expression in the muscle. These include antisense oligonucleotide–based therapies targeting hotspot *DMD* mutations between exons 45 and 53 (e.g. Exondys51, eteplirsen) and Translarna (Ataluren) which targets nonsense mutations [198, 199]. Unravelling the function and pathophysiological significance of dystrophin in the brain has become a high research priority in order to treat every element of Duchenne. A clear role for Dp71 in the neuropathogenesis of DMD is described. Further studies are required to elucidate exact mechanisms in humans, particularly regarding neurodevelopmental defects. To this end, the emergence of iPSC and organoid models will be important as well as the availability of donated human Duchenne brain tissue. Future research should also explore the interplay between Dp140 and Dp71 given the apparent neurodevelopmental origin of some DMD brain comorbidities.

RNA processing critically regulates the localisation and function(s) of Dp71 in the brain, yet we understand very little about how the *DMD* gene is regulated in this way and whether there are disruptions in Duchenne. Indeed, aberrant RNA processing is a common mechanism of disease for neurological disorders [200] and studies on the neuronal RNA processing of *DMD* may help inform ongoing drug development. Evidence shows current DMD gene therapy approaches can be used to treat the DMD brain with most studies using exon skipping to restore the reading frame and production of an internally truncated dystrophin protein [201]. For example, an AAV exon skipping vector has been delivered to the *mdx* brain resulting in improvements in hippocampal function [202]. In another study, the abnormal freezing response of *mdx* mice was rescued by intracerebroventricular delivery of an antisense oligonucleotide designed for *DMD* exon skipping [42]. Continuing improvements in the chemistry of oligonucleotides are resulting in better brain targeting, for example tricyclo-DNA antisense oligonucleotides can cross the BBB [203]. Thus whole body treatment for Duchenne is a realistic possibility, although it must be stated that distal *DMD* mutations affecting the expression of Dp71 are rare and mutation-specific treatments may have limited benefit for Duchenne patients when taken as a whole.

In summary, Dp71 is a ubiquitous and functionally diverse protein implicated in the neuropathophysiology of DMD. Advances in our understanding of its role(s) in the CNS may also have wider implications such as for cancer, neurodegeneration and neurodevelopmental and neuropsychiatric disorders.
